# Suspended soils enrich local forest floor soils during the rainy season in a tropical monsoon rainforest of Hainan Island, South China

**DOI:** 10.3389/fpls.2024.1415754

**Published:** 2024-11-07

**Authors:** Shitao Xu, Yachen Wang, Xudong Yu, Zeping Cai, Mingxun Ren

**Affiliations:** ^1^ School of Tropical Agriculture and Forestry, Hainan University, School of Agricultural and Rural Affairs, Hainan Open University, Haikou, China; ^2^ International Joint Center for Terrestrial Biodiversity around South China Sea of Hainan Province, Hainan University, Haikou, China

**Keywords:** *Asplenium nidus* L., suspended soil, canopy, monsoon rainforest, epiphytes

## Abstract

**Introduction:**

Epiphytic plants are abundant in rainforests and often serve as traps for litter and dust falling from the canopy. As it accumulates, this material can form nutrient rich soils, which are likely involved in local nutrient cycling and ecological processes.

**Methods:**

To explore spatial and temporal variation in the influence of suspended soils on local nutrient cycles, we compared the physical, chemical and biological properties of suspended soils from the locally-dominant epiphytic Bird’s nest fern (*Asplenium nidus* L.) to those of three types of forest floor soils (soil collected from upslope, downslope, and underneath the host tree) in a tropical monsoon rainforest in Bawangling National Nature Reserve on Hainan Island, China.

**Results:**

Suspended and forest floor soils were all acidic, with suspended soils having much higher organic matter (66.84%) and water content (~ 300%) than forest floor soils. Suspended soils contained significantly more available nitrogen, phosphorous, and potassium and had much higher urease, cellulase, and catalase activities, indicating that they harbored diverse microbial communities with higher decomposition and biomineralization activity.

**Discussion:**

Physicochemical traits of suspended soil and soil collected from under the host tree were significantly more similar in the rainy season than in the dry season, suggesting that suspended soils may contribute to local nutrient cycling as they are flushed out of epiphytic plants and enrich stemflow and forest floor soils.

**Conclusion:**

Thus, suspended soils play a role in local nutrient cycling, especially during the rainy season. This study provides empirical support for the seasonality and heterogeneity of forest floor soil enrichment by suspended soils in tropical monsoon rainforests.

## 1 Introduction

Canopies in tropical rainforests harbor a diversity of climbers and epiphytic plants and are distinct from the forest floor in plant richness and microclimatic conditions (Parker et al., 1995; Ozanne et al., 2003; Mafune et al., 2023). Abundant epiphytes, such as moss carpets and large ferrns (Ellwood and Foster, 2004; Beaulieu et al., 2010; Mafune et al., 2023), can accumulate dust and debris as leaves and branches fall from above (Ellwood et al., 2002). Because of the high humidity and sizeable insects, earthworms, and microbial populations that live in epiphytes’ rosettes and basket-shaped leaves (Ellwood et al., 2002; Primack, 2006; Mafune et al., 2023), this litter is often quickly decomposed into humus (Murray et al., 2023; Saeki et al., 2024). Together with newly-trapped debris and dust, this humus can be termed ‘suspended soil’ or ‘perched soil’ (Sergeeva et al., 1990; Murray et al., 2023).

Suspended soils are substantially different from forest floor soils, owing to fundamental differences in origin and composition: forest floor soils are basically mineral and suspended soils are largely organic (Cardelús et al., 2009; Beaulieu et al., 2010; Mafune et al., 2023; Murray et al., 2023). Compared to forest floor soils, suspended soils experience higher temperatures, more sunlight, and more rapid nutrient cycling and are inhabited by larger populations of invertebrates and microorganisms and considerable diversity of predatory mites (Beaulieu et al., 2010), frogs and lizards (Lindo and Winchester, 2007; Brandt et al., 2017; Saeki et al., 2024). Host trees can also affect the nutrient status, pH value and other physicochemical traits of suspended soils (Cardelús et al., 2009; Murray et al., 2023). Although the differences in nutrient content and biodiversity between forest floor and suspended soils has been studied extensively (Sergeeva et al., 1991; Ellwood et al., 2002; Hsu et al., 2002; Karasawa et al., 2008; Beaulieu et al., 2010), the relationship between suspended soils and local biogeochemical cycling and ecological processes remain largely unexplored. This is especially true in monsoon-dominated tropical Asia with pronounced drying-rewetting cycles (Ruan et al., 2004; Jiang et al., 2017).

Hainan Island is located on the northern edge of tropical Asia. It has an area of 3.4×10^4^ km^2^ and experiences summer monsoons from the Indian to the Pacific Oceans (Wang, 2002; Jiang et al., 2017). The island is home to more than 10 mountains exceeding 1500 m elevation, and all of them harbor typical tropical monsoon rainforests. A considerable number of epiphytic plants and mosses grow in these forests’ canopies, with *Asplenium nidus* L. (Aspleniaceae) dominates the epiphytic niche. Suspended soils are found in this species’ basket-shaped rosettes, but it can be easily washed out by heavy rains, flowing down the trunk to the ground (S.T.X. & M.X.R., personal observation). This phenomenon led us to hypothesize that the nutrients contained in suspended soils may infiltrate the forest floor during the rainy season, influencing local ecological processes and biogeochemical cycling.

The aims of the present study were (i) to quantify the physical, chemical, and microbial properties of suspended soils accumulating in and on bird’s nest ferns (*Asplenium nidus* L.) in Bawangling National Nature Reserve on Hainan Island; (ii) to quantify differences in soil physicochemical properties between suspended and forest floor soils; and (iii) to explore the possible seasonal and heterogeneous contributions of suspended soils to local nutrient cycles. The results will contribute to a better understanding of these soils’ ecological functions and the dynamics of their fundamental properties in tropical monsoon rainforests.

## 2 Materials and methods

### 2.1 Study site

The study area was located at Bawangling Nature Reserve on Southwest Hainan Island (109° 03’-17’E, 18° 57’-19° 11’N). The reserve covers an area of 29,980 ha with elevation ranging from 100 m to 1560 m. The climate is dominated by typical summer monsoons, with distinct rainy (May-October) and dry seasons (November-March). Mean annual rainfall is 2000 mm, with most precipitation falling during heavy rainstorms during the rainy season (Ding and Zang, 2005). Relative humidity is usually above 80% (Ding and Zang, 2005; Chen et al., 2009). Such weather results mainly from tropical monsoons from the Indian and northwest Pacific Oceans (Wang, 2002; Jiang et al., 2017).

**Figure 1:**
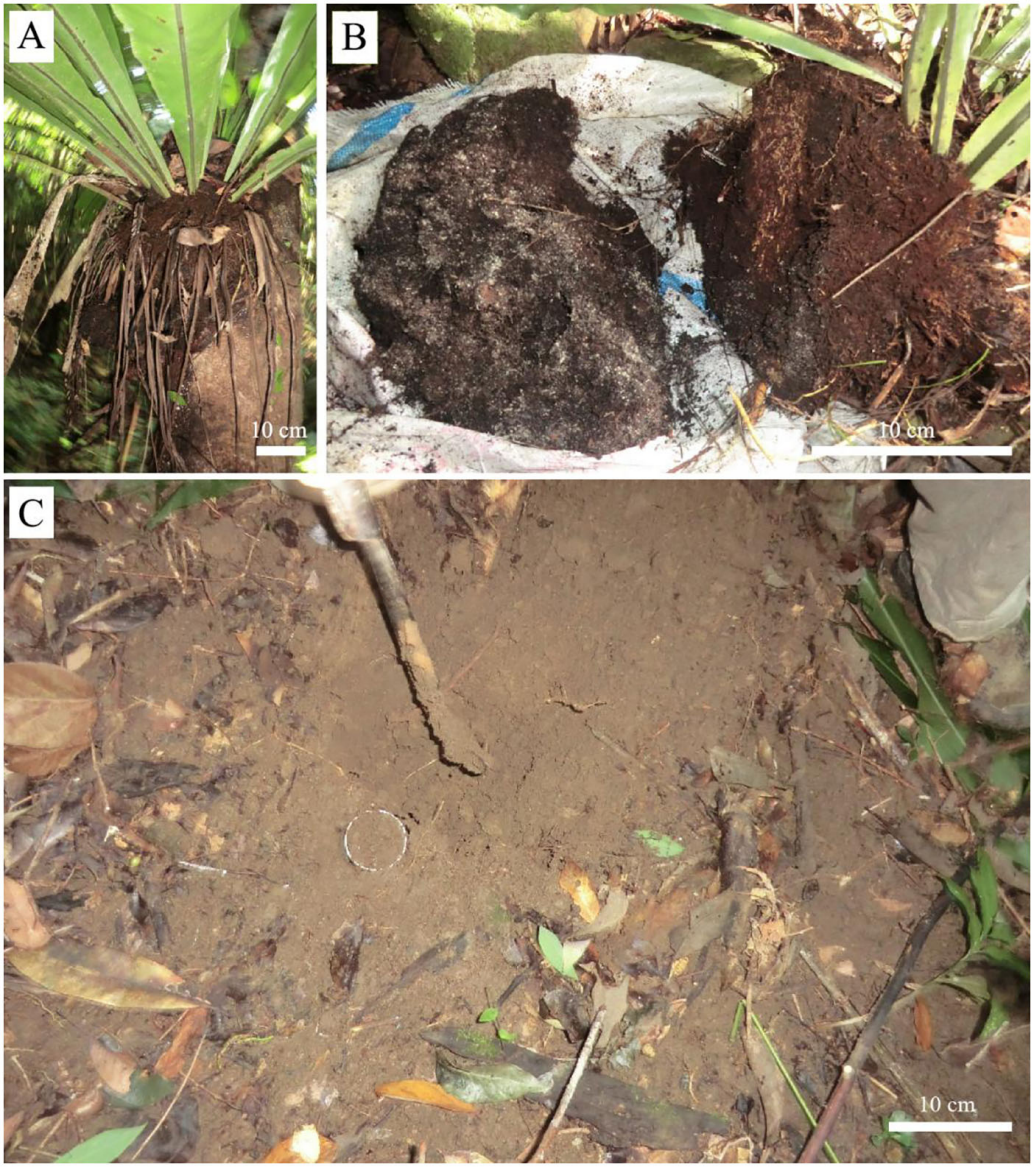
Suspended soils **(A, B)** and forest floor soils **(C)** in the study site.

*A. nidus* predominates the epiphytic habitat in slopes along the banks of the biggest river on the island, the Nancha River. The slopes angle downward at about 10 degrees, with dense latosol soil on the floor (**Figure 1**). The dominant trees are *Wendlandia uvariifolia*, *Ficus laevis*, *Nyssa sinensis*, *Bischofia polycarpa*, *Glochidion wrightii*, and *Baccaurea ramiflora* (Ding and Zang, 2005; Xu, 2013).

### 2.2 Sample collection

Adult individuals (basal diameter of the rosette > 0.5 m) perched on tree trunks were sampled in March (dry season) and August (rainy season) of 2016 along two slopes along the Nancha River. A minimum of 500 g of suspended soil was collected from each rosette and stored in its own plastic bag. In total, suspended soils from 35 and 38 A*. nidu*s individuals were collected for the dry and rainy seasons, respectively (**Figure 1**).

We collected samples of forest floor soil from under trees hosting sampled bird’s nest ferns. We collected a second forest floor sample from a location ~3 m upslope of the host tree and a third ~3 m downslope. At each sampling location, debris was removed from the surface and five replicates were collected, each to a depth of 10 cm. Samples were divided into two portions using 6.5 mm and 2 mm sieves. Root fragments were removed from the > 6.5 mm samples. A portion of each sample was reserved for later measurements of microbial biomass C, N, and respiration and placed in coolers to the field prior to transport to the laboratory. Samples to be analyzed for physicochemical properties and microbial activity were stored in cotton bags prior to processing (drying, grinding, sorting into size classes, and bottling).

### 2.3 Soil analysis

We subjected suspended and forest floor samples to chemical and physical analysis as well as microbial and enzymatic assays. The organic matter content of soils was determined by wet-oxidation using potassium dichromate with external heat and back-titration (Zhang and Gong, 2012). Available P and K concentrations were determined via Mo-Sb anti-spectrophotometry and the inductively coupled plasma atomic emission spectrometer (ICP-AES) method.

Total P and K were quantified using ICP-AES after treatment with HClO_4_. Total N was measured using the Kjeldahl digestion method. Nitrate N and ammonium N were determined by colorimetric spectrophotometry using indophenol blue and phenol disulfonic acid. Soil pH was measured using a 25:1 soil:water ratio (Zhang and Gong, 2012).

Soil microbial biomass was measured using the chloroform fumigation-incubation method and a conversion factor of 0.45. Soil respiration was determined by the alkali trapping method. Soil urease activity, cellulase activity, sucrose content, nitratase activity, and polyphenol oxidase activity were assessed colorimetrically using sodium phenolate (Wu et al., 2012), anthronem, 3,5-dinitro salicylic acid (Xu and Zheng, 1986), disodium phenyl phosphate (Zhao and Jiang, 1986), and pyrocatechol (Xu and Zheng, 1986), respectively. The sodium permanganate titration method was used to measure catalase activity (Wu et al., 2012).

### 2.4 Data analysis

Excel 2007 and SPSS 20.0 were used for data input and statistical analysis. The traits of suspended soil and all three types of forest floor soil were compared using histograms generated in Excel 2007. Differences between soil types were compared using one-way ANOVA on ranks, followed by a Tukey’s test for all multiple pairwise comparisons. All statistical analyses were performed using SPSS 20.0 (SPSS, Chicago, U.S.A.)

We used detrended correspondence analysis in CANOCO version 4.5 to explore relationships between soil nutrients and environmental factors. Redundancy analysis was then performed using CANOCO and graphs produced in CanoDraw to determine the main environmental factors affecting soil nutrient status. All data were log-transformed.

To visualize similarity and clustering patterns between suspended and forest floor soil, we generated a matrix plot of all 16 soil traits considered using PAST Version 3 (Hammer et al., 2001). We also created a dendrogram based on UPGMA (Unweighted Pair Group Method with Arithmetic Mean) relationships between the four sample types.The 16 soil traits are water content (%), pH, total N, available K, available P, organic matter (%), urease, cellulose, sucrose, nitratase, catalase, polyphenol oxidase, respiration intensity, bacterial content, fungal content, and *Actinomycetes* content. These variables are coded from A to P, respectively, in the data analyses.

## 3 Results

### 3.1 Soil traits in the dry season

**Figure 2:**
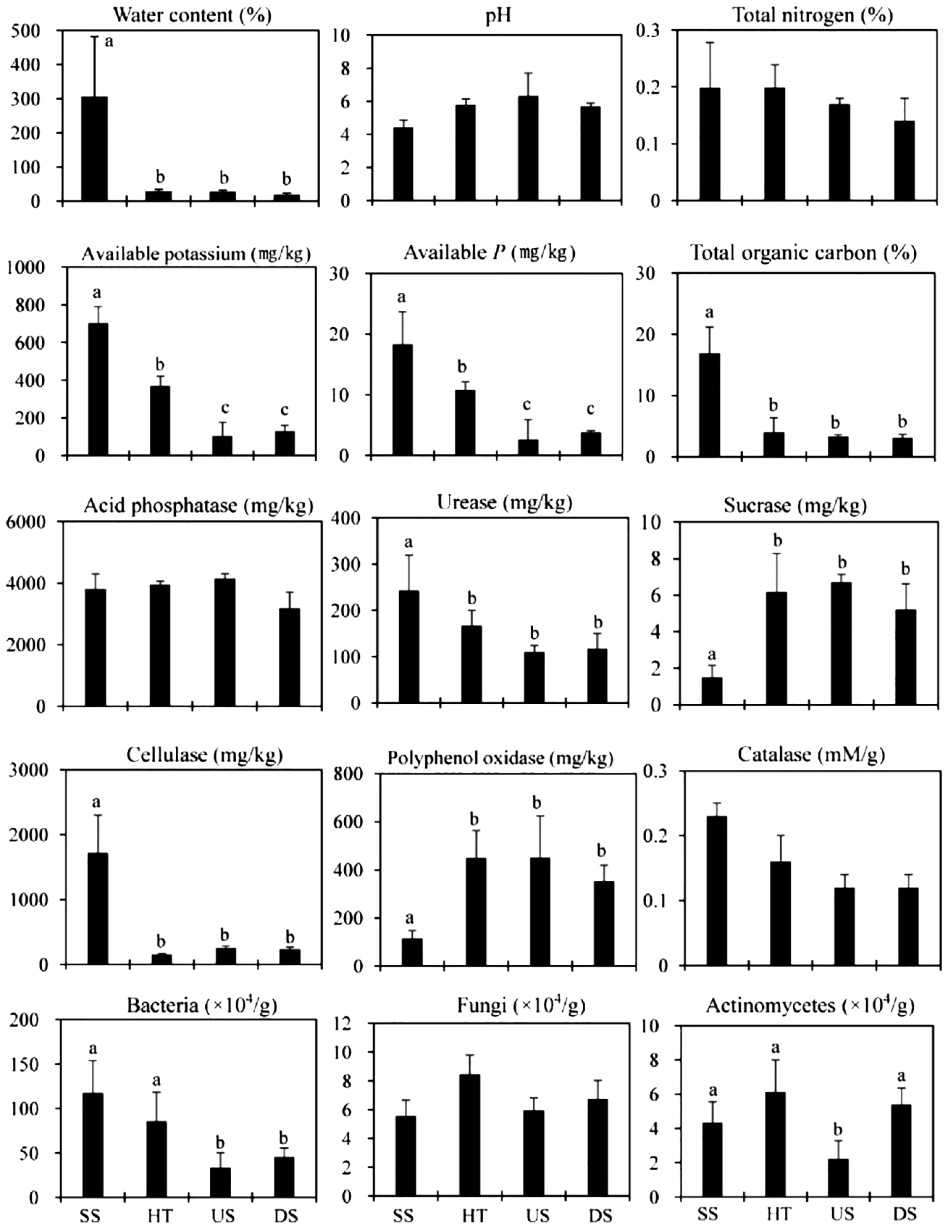
Soil nutrient status, enzyme activity and microbial characteristics in the dry season. SS, Suspended soils; HT, Soil under host tree; US, Soil from 3 m upslope of the host tree; DS, Soil from 3 m downslope of the host tree. Different letters indicate significant differences (*P* < 0.05).

Suspended soil had much higher water, organic matter, and available K and P content than forest floor soils (**Figure 2**). The moisture content of suspended soil was especially high at 306%, indicating a large water holding capacity. Organic matter content was also dramatically different between suspended and forest floor soil, with forest floor soil collected from under host trees and upslope/downslope containing 17 and 27 times less organic matter than suspended soils, respectively (**Figure 2**).

No significant differences in pH or total N were detected between suspended and forest floor samples (**Figure 2**). For forest floor samples, soil collected from under host trees had significantly more available P and K relative to upslope and downslope soils (**Figure 2**).

Suspended soils had more bacterial, Actinomycetes, and fungal colonies than forest floor soils. We also observed higher respiratory activity in suspended soils (**Figure 2**). All differences were highly significant (P<0.01) under one-way ANOVA analyses. Soil enzymes exuded by microorganisms (*i.e.*, urease, cellulase, and catalase) were also much higher in suspended soils (**Figure 2**). Forest floor soils had higher sucrase and polyphenol oxidase activities than suspended soils (**Figure 2**).

### 3.2 Soil traits in the rainy season

**Figure 3:**
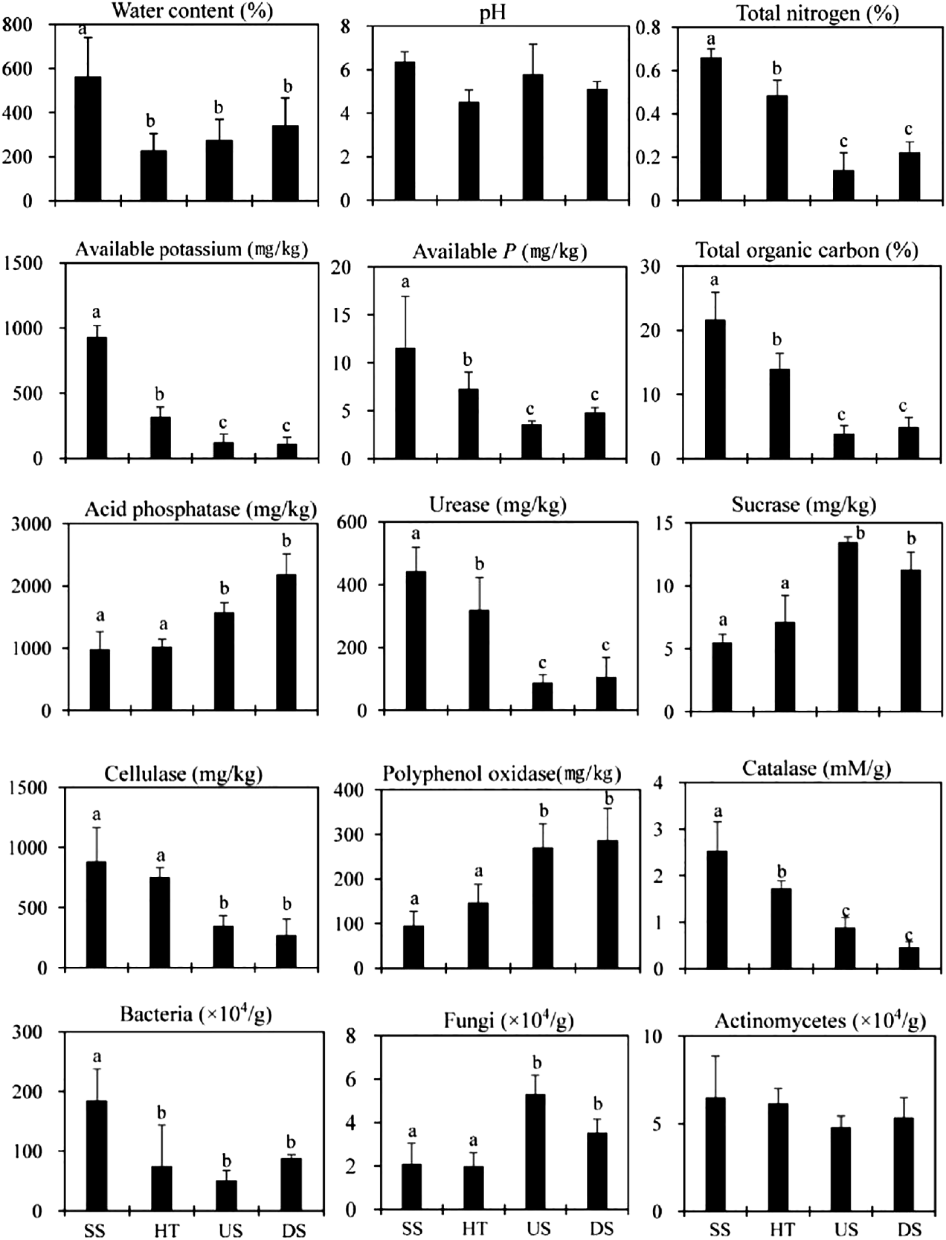
Soil nutrient status, enzyme activity and microbial characteristics in the rainy season. SS, Suspended soils; HT, Soil under host tree; US, Soil from 3 m upslope of the host tree; DS, Soil from 3 m downslope of the host tree. Different letters indicate significant differences (*P* < 0.05).

We observed similar patterns in soil traits across seasons (**Figure 3**). However, soil collected from under host trees was more similar to suspended soils than to samples collected up- or down-slope (**Figure 3**), with all traits intermediate between values observed in suspended soil and those observed in up- or down-slope soil. There was no significant difference between suspended and forest floor soils for acid phosphatase, sucrase, cellulose, catalase, and polyphenol oxidase activities (**Figure 3**), indicating that the composition and activity of soil microbial communities were similar between these two host tree-connected soils.

**Figure 4:**
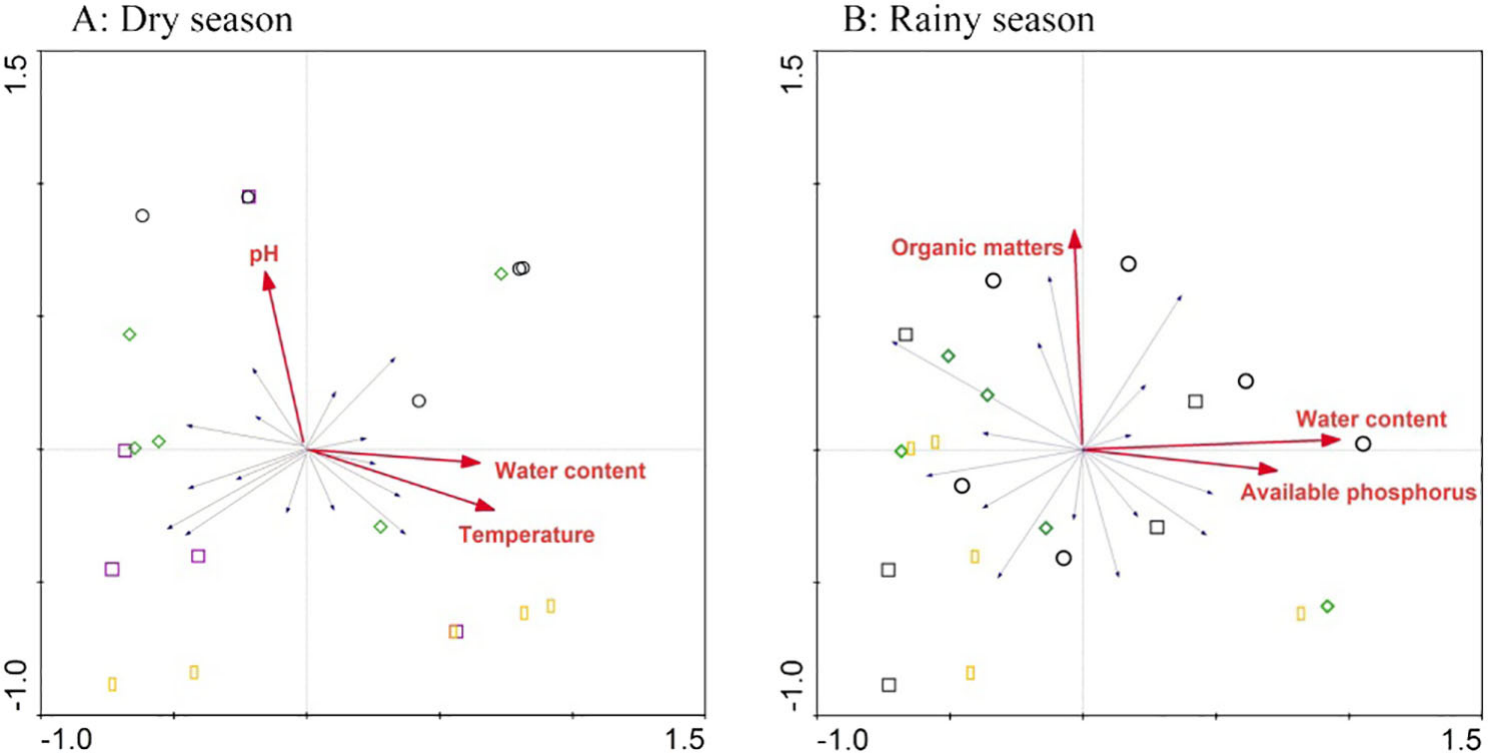
Biplot of the first two axes of the redundancy analysis for environmental factors associated with soil nutrients in the dry **(A)** and rainy **(B)** seasons, respectively.

Redundancy analysis results revealed that the main environmental factors affecting soil nutrient status and microbial activity were pH, water content, and temperature in the dry season. In the rainy season, organic matter content, moisture content, and available P had the strongest influence (**Figure 4**).

### 3.3 Comparison between soil types in the rainy and dry seasons

**Figure 5:**
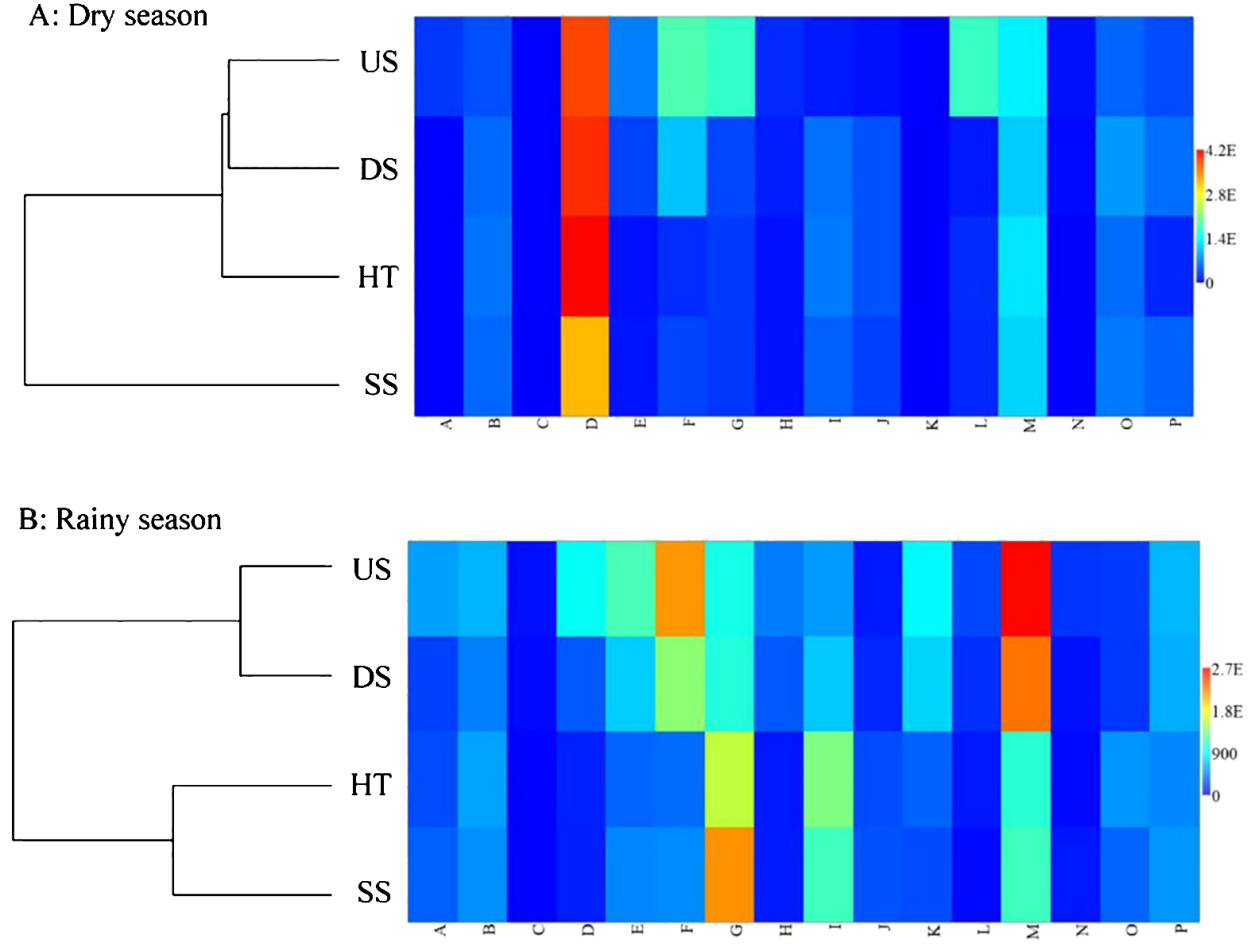
Dendrogram based on UPGMA relationship with similarity plotting of all 16 soil traits using PAST 3 for suspended soils (SS) and three types of forest floor soils (HT, US, DS) in dry **(A)** and rainy **(B)** seasons.

Our clustering analysis and matrix plot showed a clear distinction in nutrient content and microbiological traits between suspended and forest floor soils in the dry season. Specifically, the suspended soils showed different soil traits as compared with all the three types soils, i.e. soils under the host tree (HT) and soils from 3 m upslope of the host tree (US) and soils from 3 m downslope of the host tree (DS) (**Figure 5A**). In contrast, increased similarity between the two soil types, i.e. SS and HT, was observed in the rainy season (**Figure 5B**) and both showed higher contents of organic matter (*G* in **Figure 5B**) and total nitrogen (*I* in **Figure 5B**) as compared to US and DS soils.

## 4 Discussion

Suspended soil had much higher water content than forest floor soil in both the dry and rainy seasons (**Figures 2**, **3**), suggesting that fern rosettes and suspended soils are always saturated. This is consistent with work done in Sabah, Malaysia by Turner and Foster (2006), who found that bird’s nest ferns can intercept rainfall and whose sponge-like root masses are always saturated with water. Compared to suspended soils, forest floor soils are more prone to losing water due to slope drainage, especially during the dry season (**Figures 2**, **3**). More importantly, we also found that suspended soils had much higher organic matter and nutrient content than forest floor soils, signifying their ability to trap, transform, and absorb nutrients. This is consistent with several prior studies (Sergeeva et al., 1991; Couto-santos and Luizao, 2010). The fern’s large, basket-shaped rosette functions to collect litterfall, particles suspended in the atmosphere, and the decaying biomass of microbes and invertebrates that inhabit the plant (Ellwood and Foster, 2004; Beaulieu et al., 2010). Under high humidity and temperatures and high oxygen content, these materials are transformed into humus easily via interactions between the fern’s roots and organisms that inhabit the plant. The lower pH of suspended soil in the dry season (**Figure 2**) may be due to the formation of organic and inorganic acids during litter decomposition associated with invertebrates and microorganisms (Brady, 1990; Cardelús et al., 2009; Mafune et al., 2023; Saeki et al., 2024). In a old-growth forest with huge Japanese cedars (*Cryptomeria japonica*) (Saeki et al., 2024) and with *Acer macrophyllum* trees (Mafune et al., 2023), soil invertebrate communities in suspended soils were found to be more diverse and distinct from those on the forest floor. Such phenomena can be a reason for the observed lower pH of suspended soils in our study.

For soil microorganisms, bacteria were more abundant in suspended verusu forest floor soils in both dry and rainy seasons (**Figures 2**, **3**), suggesting relative higher nutrient contents in the suspended soils. On the other hand, soil fungi showed no significant difference between soil types in dry season, but higher content of soil fungi in forest floor soils was observed in rainy season (**Figures 2**, **3**). These findings suggested that soil microorganisms changed notably during the dry and rainy dynamics and the forest floor soils might be affected by suspended soils. Suspended soils, compared to forest floor soils, were a pool of unique microbiome biodiversity (Eskov et al., 2021). The main driving factor was the dominant organic matter in suspended soils collected by the epiphytes and degraded by annelid, termites and other soil fauna. Furthermore, The humification process occurred in suspended soils was found with the absence of mineral compounds or mineral fine earth (Abakumov et al., 2018), indicating humification associated with pure organic substrates can result in formation of deep humified organic matter (Abakumov et al., 2018). Soil nutrient content might be a driving factor for soil respiration activity and microbial biodiversity (Eskov et al., 2021). Such patterns in nutrient content, soil respiration activity and microbial biodiversity of suspended soils was observed in dry season (**Figure 2**) and rainy season (**Figure 3**).

Rosettes and the suspended soil they hold therefore form a treetop ecological “island,” providing a distinctive habitat for various invertebrates (Saeki et al., 2024), microbes (Sergeeva et al., 1991; Mafune et al., 2023), and even some frogs, lizards and predatory mites (Beaulieu et al., 2010; Couto-santos and Luizao, 2010). The microhabitat and resource patches created by the bird’s nest fern offer nutrient enrichment services to the forest ecosystem by internalizing external nutrient resources (i.e., atmospheric particles nutrient condensation) as well as by trapping litterfall. Thus, the large basket-shape bird’s nest fern, with its soil system, acts as a visible ‘mid-canopy’ and serves important ecosystem functions, including promoting species diversity, enriching and condensing nutrients, and cycling water. Similar patterns have been identified in Central and North America (Nadkarni and Matelson, 1992), south Vietnam (Abakumov et al., 2018; Eskov et al., 2021) and the tropical regions of Yunan Province, southwest China (Wang et al., 2008). Our findings were basically consistent with other studies in tropical America and Africa and thus proved that suspended soils in the rainforest canopy palyed significant role in the local nutrient cyclings, not only on the top of the rainforest but also in the forest floor soil ecosytem.

The increase in similarity between suspended and forest floor soil traits in the rainy season (**Figure 5**) suggests that rainfall might drive stemflow along the host tree by flushing suspended soils out of rosettes, enriching forest floor soil. This demonstrates that the spatial and temporal alternation of local nutrient distribution is caused by seasonal rainfall or by host-epiphyte detachment in rainforests characterized by drying-rewetting cycles. In a subtropical evergreen broad-leaved forest in southeast China, Li and Lin (1998) also found that stemflow deposited a considerable quantity of nutrients to forest floor soil. Therefore, suspended soils may modulate local biogeochemical cycles by enriching host tree stemflows and fertilizing forest floor soils. This may be important for ecosystem functioning and biodiversity maintenance in tropical monsoon rainforests with drying-rewetting cycles that alter the spatiotemporal patterns of heterogeneous habitats associated with suspended soils (Ruan et al., 2004; Levia and Frost, 2006). Epiphytes and their suspended soils can alter stemflow nutrient concentrations by slowing water percolation and by nutrient uptake and release, affecting nutrient recyclings between tree conapy and forest floor ecosystem (Strigel et al., 1994; Awasthi et al., 1995; Victoriano-Romero et al., 2020) and imparting a resiliency to disturbances via providing “nutrient reserve” for local ecosystems (Mafune et al., 2023).

Turner et al. (2007) pointed out that stemflow is enriched by prolonged contact and nutrient deposits on the trunk (Liu et al., 2002). These nutrient deposits are formed largely by the suspended soils that are washed out of epiphytic plants. The higher nutrient concentration in the stemflow of host trees with epiphytes has important localized effects that contribute to the spatial heterogeneity of forest ecosystems via altering nutrient availability in the immediate area around host trees (Crozier and Boerner, 1984; Victoriano-Romero et al., 2020). In tropical monsoon rainforests, the dominant drying-rewetting cycles caused by monsoons probably further intensify such seasonality and heterogeneity of local nutrient cycles. In south Veitnam, Abakumov et al. (2018) found that the pH of suspended soils was lower that forest floor soils, while basal respiration did not change between two soil typesder phorophytes and suspended soils and the suspended soil was less enriched by nitrogen than soils from forest floor. Similar pattern was observed in our study, especially in the rainy season (**Figure 2** and **Figure 3**). This can be related to the total amount of organic matter exposed to humification in various soils and to the presence of an essential portion of mineral particles in the forest floor soils (Abakumov et al., 2018). The humic acids of forest floor soils showed the same content of aromatic fraction as the suspended soils. The humification process implemented in suspended soils showed the absence of mineral compounds or mineral fine earth, suggesting that humification with pure organic substrates can facilitate the formation of deep humified organic matter (Abakumov et al., 2018; Eskov et al., 2021).

In this study, we demonstrated that suspended soils from epiphytes may enrich soils under the host trees and increase the seasonality and heterogeneity of local nutrient cycles in a monsoon-dominated rainforest with a well-defined drying-rewetting cycle. Such differential responses in ecosystem functioning at both the spatial and temporal scales may leave monsoon rainforests more vulnerable to habitat conversion and climate change than other systems. Future studies about the ecology and conservation dynamics of tropical monsoon rainforests should pay more attention to the seasonal dynamics of local nutrient cycling and their effects on the spatial heterogeneity of resources and nutrients.

## Data Availability

The original contributions presented in the study are included in the article/supplementary material. Further inquiries can be directed to the corresponding author.
